# Multidisciplinary Care Model as a Center of Excellence for Fabry Disease: A Practical Guide to Diagnosis and Management by Clinical Specialty in South Korea

**DOI:** 10.3390/jcm14134400

**Published:** 2025-06-20

**Authors:** Soo Yong Lee, Il Young Kim, Sung-Ho Ahn, Su Jin Kim, Hyun-Min Lee, Ji Eun Lee, Gyeong-Jo Byeon, Hyun-Chang Ko, Hyun Jung Lee, Songhwa Choi, Chong Kun Cheon

**Affiliations:** 1 Division of Cardiology, Department of Internal Medicine, Pusan National University Yangsan Hospital, Pusan National University School of Medicine, Yangsan 50612, Republic of Korea; shonge0906@pusan.ac.kr; 2 Research Institute for Convergence of Biomedical Science and Technology, Pusan National University Yangsan Hospital, Yangsan 50612, Republic of Korea; iykim@pusan.ac.kr (I.Y.K.);; 3 Division of Nephrology, Department of Internal Medicine, Pusan National University Yangsan Hospital, Pusan National University School of Medicine, Yangsan 50612, Republic of Korea; 4 Department of Neurology, Pusan National University Yangsan Hospital, Pusan National University School of Medicine, Yangsan 50612, Republic of Korea; 5 Division of Gastroenterology, Department of Internal Medicine, Pusan National University Yangsan Hospital, Pusan National University School of Medicine, Yangsan 50612, Republic of Korea; 6 Department of Otorhinolaryngology-Head and Neck Surgery, Pusan National University Yangsan Hospital, Pusan National University School of Medicine, Yangsan 50612, Republic of Korea; 7 Department of Ophthalmology, Pusan National University Yangsan Hospital, Pusan National University School of Medicine, Yangsan 50612, Republic of Korea; 8 Department of Anesthesia and Pain Medicine, Pusan National University Yangsan Hospital, Pusan National University School of Medicine, Yangsan 50612, Republic of Korea; 9 Department of Dermatology, Pusan National University Yangsan Hospital, Pusan National University School of Medicine, Yangsan 50612, Republic of Korea; 10 Department of Pathology, Pusan National University Yangsan Hospital, Pusan National University School of Medicine, Yangsan 50612, Republic of Korea; 11 Medical Affairs, Takeda Pharmaceuticals Korea Co., Ltd., Seoul 05551, Republic of Korea; 12 Division of Medical Genetics, Department of Pediatrics, Pusan National University Children’s Hospital, Pusan National University School of Medicine, Yangsan 50612, Republic of Korea

**Keywords:** Fabry disease, diagnosis, treatment, practice guideline, multidisciplinary team approach

## Abstract

Fabry disease (FD) is a lysosomal storage disorder caused by pathogenic variants in the gene encoding alpha-galactosidase A (GLA). Deficiency of GLA results in the progressive accumulation of glycosphingolipids in virtually all organs, resulting in a progressive multisystem disease. Due to multi-organ involvement in FD, a comprehensive, multidisciplinary approach to diagnosis and treatment with regular follow-ups is essential. The Pusan National University Yangsan Hospital (PNUYH) multidisciplinary care model of FD aims to provide detailed practice guidelines and evidence-based recommendations for the diagnosis, screening, and treatment of FD according to specialty. This guideline focuses on the “quarterback” type of multidisciplinary team (MDT) operation and is limited in its applicability to the Korean insurance system. However, it reflects our team’s extensive experience and insights into optimizing MDT operations within these constraints and is expected to be highly beneficial for centers initiating MDTs for the effective treatment of FD.

## 1. Introduction

Fabry disease (FD) is a lysosomal storage disorder caused by pathogenic variants in the gene encoding alpha-galactosidase A (GLA) [1]. Deficiency of GLA results in the inability of cells to catabolize glycosphingolipids with terminal α-D-galactosyl residues [2]. Glycosphingolipids, particularly globotriaosylceramide (Gb3), progressively accumulate in virtually all organs, resulting in a progressive multisystem disease. The cardinal features of the disease are acroparesthesia (burning pain in hands and feet), progressive proteinuric renal insufficiency, and cardiac disease consisting of rhythm and conduction disturbances and progressive cardiac hypertrophy, as well as cerebrovascular stroke.

FD is characterized by gradually deteriorating functions over time as the disease invades various organs of the body. The involvement of vital organs such as the heart, kidneys, and brain leads to critical and potentially fatal complications, including organ failure and stroke. Furthermore, peripheral neuropathies and non-specific symptoms, such as gastrointestinal or otorhinolaryngological symptoms, may significantly impair the quality of life of patients with FD.

A multidisciplinary team (MDT) approach to treatment involving experts in various fields is crucial for managing FD, considering its complexity, rarity, and multi-systemic nature. The MDT approach is important for an accurate and comprehensive diagnosis, personalized and holistic care, improved coordination and efficacy, enhanced collaboration and innovation, and to address systemic challenges [3,4]. It also ensures patient access to cutting-edge treatments while reducing the burden on families. This collaborative treatment model is essential for improving outcomes in a field where individual experts alone often fall short.

For the first time, a Fabry center of excellence was started in 2021 by the Pusan National University Yangsan Hospital (PNUYH) in Korea to discuss challenging patient cases with diverse clinical courses of FD. Since then, specialists from cardiology, nephrology, neurology, ophthalmology, dermatology, otolaryngology, pathology, pain medicine, digestive medicine, and psychiatry have joined the team for in-depth discussion of research and case studies of FD and to collate their experience and insights into a management guideline for Korean patients with FD.

This guideline aims to provide practical guidance for an MDT approach to the treatment of FD and to improve communication between experts involved in diagnosing and treating FD. Recommendations in the guideline are based on comprehensive literature reviews and clinical treatment experiences. For specialties where sufficient data or evidence regarding the diagnosis and treatment of FD is not available, the level of recommendations is not specified.

**Summary of Key Recommendations according to Clinical Specialty**

**Genetic Medicine**

*Diagnosis*

Early diagnosis through enzyme activity testing and genetic analysis is recommended.Combine insights from disease-specific databases and in vitro prediction tools with expert clinical opinion, defining the relative weight of each American College of Medical Genetics and Genomics criterion.Review the literature and databases periodically to check whether a variant has been reclassified.
*Treatment and follow-up*
Regular monitoring of organ function and biomarkers is essential.A comprehensive, multidisciplinary approach (nephrologist, cardiologist, neurologist, dermatologist, gastroenterologist, ophthalmologist, anesthesiologist, and otolaryngologist) to treatment with regular follow-ups should be used, integrating knowledge from multiple disciplines to diagnose and address any arising problems.

**Cardiology**

Measurement of plasma B-type natriuretic peptide (BNP)/NT-proBNP is recommended in symptomatic patients with suspected heart failure (Class I, Level of Evidence B).Standard 12-lead electrocardiography (ECG) and 2D echocardiography are recommended at initial evaluation and on the development of new symptoms. A follow-up of around 12 months is reasonable (Class I, Level of Evidence B).Cardiac magnetic resonance imaging, including native T1 mapping and contrast-enhanced T1 mapping, may be considered every 5 years in adult patients to assess the progression of fibrosis and left ventricular function (Class IIb, Level of Evidence C).Endomyocardial biopsy can be considered in patients with variants of unknown significance in the alpha-galactosidase A (*GLA)* gene and normal residual GLA activity (>10%) to confirm organ involvement of Fabry disease (FD) (Class IIa, Level of Evidence C).Aim for normal blood pressure (BP; <130/80 mmHg) in all patients according to current hypertension management guidelines (Class IIa, Level of Evidence C).The risk of cardiovascular disease in FD can be considered similar to that of diabetes. Therefore, the target level of low-density lipoprotein (LDL) cholesterol for each patient should be determined by the disease duration and accompanying risk factors (Class IIb, Level of Evidence C).

**Nephrology**

Early screening for Fabry nephropathy in high-risk populations may be beneficial, including in patients with unexplained proteinuria or chronic kidney disease of unknown origin, and in relatives of diagnosed patients with FD (Class II, Level of Evidence C).A combination of diagnostic tools, such as GLA enzyme activity measurement, genetic testing, and plasma globotriaosylsphingosine levels, can be considered to confirm Fabry nephropathy and guide initial management (Class II, Level of Evidence C).Early initiation of enzyme replacement therapy in patients with Fabry nephropathy may help to stabilize renal function and prevent progression to end-stage renal disease (Class II, Level of Evidence C).Additional therapies, including renin–angiotensin–aldosterone system inhibitors and newer agents like sodium-glucose cotransporter-2 inhibitors, may offer advantages in managing proteinuria and improving renal outcomes in Fabry nephropathy (Class II, Level of Evidence C).Regular monitoring of renal biomarkers, including proteinuria, albuminuria, serum creatinine, and estimated glomerular filtration rate, can assist in evaluating disease progression and therapeutic response (Class II, Level of Evidence C).Structured follow-up visits every 3 to 6 months, along with an annual comprehensive review, may enable timely adjustment of therapy and optimize patient outcomes (Class II, Level of Evidence C).
**Neurology** Patients with FD are at high risk for atherosclerotic cardiovascular disease and should follow a comprehensive stroke prevention strategy, especially in ischemic stroke or transient ischemic attack (TIA). Recommendations for secondary prevention of stroke follow American Heart Association/American Stroke Association (AHA/ASA) guidelines. *Management of risk factors for stroke* For hypertensive patients, target office BP < 130/80 mmHg (Class I, Level of Evidence B).For stroke/TIA with atherosclerosis, statins ± ezetimibe to lower LDL < 70 mg/dL are advised (Class I, Level of Evidence A).For diabetic patients, aim for HbA1c ≤7%, individualized to age and comorbidity (Class I, Level of Evidence A).*Management of intracranial large artery atherosclerosis* For 50–99% stenosis, aspirin 325 mg/d is preferred over warfarin (Class I, Level of Evidence B).For recent stroke/TIA (<30 days) and 70–99% stenosis, add clopidogrel 75 mg/d to aspirin for up to 90 days (Class IIa, Level of Evidence B).Aim for systolic BP < 140 mmHg, use high-intensity statin, and encourage physical activity (Class I, Level of Evidence B).*Management of extracranial large artery atherosclerosis* For TIA or minor stroke with 70–99% carotid stenosis within 6 months, carotid endarterectomy (CEA) is recommended if the perioperative risk is <6% (Class I, Level of Evidence A).CEA or stenting should be performed by experienced operators (<6% perioperative risk) (Class I, Level of Evidence A).Intensive medical therapy (antiplatelets, statins, BP control) is advised for all patients with carotid stenosis and TIA/stroke (Class I, Level of Evidence A).Same intensive therapy is also advised for patients with recent symptomatic extracranial vertebral artery stenosis (Class I, Level of Evidence A).*Management of small vessel disease* The benefit of cilostazol in small vessel stroke is uncertain (Class IIb, Level of Evidence B).*Management of cardioembolism* For nonvalvular atrial fibrillation (AF) with stroke/TIA, oral anticoagulants (e.g., direct oral anticoagulants or warfarin) are recommended (Class I, Level of Evidence A).For valvular AF (e.g., mitral stenosis, mechanical valve), warfarin is indicated (Class I, Level of Evidence B).In stroke/TIA with native aortic or nonrheumatic mitral valve disease and no AF, antiplatelets are recommended (Class I, Level of Evidence C).In patients with a bioprosthetic valve with prior stroke/TIA, long-term aspirin is preferred over anticoagulation 3–6 months post-surgery (Class I, Level of Evidence C).
**Gastroenterology**

Gastroenterologists should be aware of FD as a possible cause of non-specific gastrointestinal symptoms (Class II, Level of Evidence B).Certain medications can provide symptom improvement for specific gastrointestinal symptoms (Class II, Level of Evidence C).

**Otorhinolaryngology**

Conduct comprehensive initial assessments for all FD patients, including thorough medical history taking and systematic symptom evaluation using validated questionnaires.Perform age-appropriate diagnostic testing: For patients aged more than 6 years, conduct pure-tone audiometry (PTA), speech audiometry (SA), tympanometry, and otoacoustic emissions (OAE); for children under the age of 6 years, perform distortion product OAE (DPOAE) initially, followed by auditory brainstem response (ABR) and auditory steady-state response (ASSR) if abnormalities are detected.Implement annual hearing assessments for all patients with FD, regardless of age or symptom status, including PTA, SA, DPOAE, and tympanometry. For pediatric patients, conduct age-appropriate hearing tests at least annually.Administer vestibular function tests for patients reporting dizziness or vertigo and perform tinnitogram for those with tinnitus complaints.Provide appropriate interventions for hearing loss, tinnitus, and vertigo based on individual patient needs, and consider hearing aids for hearing loss and cochlear implants for severe cases.Treat sudden sensorineural hearing loss and acute vertigo as medical emergencies requiring immediate intervention and establish a prompt and effective interdisciplinary consultation system for acute symptoms.

**Ophthalmology**

Corneal manifestations: The hallmark corneal finding in FD is corneal verticillata, also referred to as vortex keratopathy. This is characterized by grayish or golden-brown deposits in the corneal epithelial and subepithelial layers, arranged in a distinctive vortex pattern. Conjunctival findings: In the conjunctiva, round intracellular inclusions are often present, accompanied by vascular abnormalities such as tortuosity, microaneurysms, and dilated blood vessels.Lens opacities: Cataract may occur in FD, typically presenting as anterior capsular or posterior subcapsular opacities.Retinal and optic nerve changes: Retinal vascular tortuosity is frequently observed in patients with FD, although optic disc edema is rarely seen. These retinal findings can provide additional diagnostic clues.Corneal surgery considerations: Although most corneal lesions in FD are non-progressive, careful consultation is advised before performing corneal refractive surgery. There is a potential risk of progression of corneal opacity postoperatively, necessitating thorough preoperative evaluation.

**Pain Management**

Acute pain medications among patients with FD include non-steroidal anti-inflammatory drugs (NSAIDs) and non-opioid analgesics, with few patients requiring neuropathic pain-control agents.Chronic pain management in FD relies on clinical experience and guidelines for neuropathic pain. First-line options include tricyclic antidepressants, serotonin and norepinephrine reuptake inhibitors, carbamazepine, and gabapentinoids. A multidisciplinary approach is important when forming an analgesic care plan, involving not only metabolic and pain specialists but also specialist pharmacists, pain psychologists, and physiotherapists.



## 2. Diagnosis and Follow-Up Assessments

### 2.1. Genetics

Effective diagnosis and follow-up of FD in a medical genetic department requires a comprehensive multidisciplinary approach. Early diagnosis through enzyme activity testing and genetic analysis, combined with regular monitoring of organ function and biomarkers, is essential for optimal patient management. By implementing a structured follow-up protocol, clinicians can reliably track disease progression, adjust treatments, and improve the quality of life of patients with FD.

FD manifests with a wide spectrum of symptoms, including acroparesthesia, angiokeratoma (small, dark red spots on the skin), hypohidrosis (decreased ability to sweat), corneal opacity, gastrointestinal issues, tinnitus and hearing loss, progressive kidney failure, heart problems, and stroke. Symptoms occur widely across the body, necessitating a multidisciplinary approach to recognizing and managing them.

The diagnostic approach for FD is gender-specific due to its X-linked inheritance pattern. The diagnostic algorithm can be summarized as follows:

For males:

1. Measure GLA enzyme activity in blood, with GLA activity < 1% being highly suggestive of classic FD.
2. Genetic testing involves sequencing the *GLA* gene to confirm the diagnosis.

For females:

1. Genetic testing by sequencing the *GLA* gene is mandatory.
2. Measurement of GLA enzyme activity is optional, as the activity may be within the normal range due to random X-chromosome inactivation.

Assessment of biomarkers such as globotriaosylsphingosine (lyso-Gb3) is important for identifying atypical FD variants. High-sensitive troponin T (hsTNT) may be useful for identifying cardiac involvement.

The Korean reimbursement system provides the following diagnostic criteria for initiating enzyme replacement therapy (ERT) (Table 1). In our center, patient eligibility for treatment is verified based on these criteria and the following tests are conducted to accurately assess the involvement of various organs.

Initial evaluation of FD should include the following items:

- Comprehensive medical history and physical examination.
- Family history assessment.
- Baseline organ function tests:
  ✓ Renal function: proteinuria, microalbuminuria, estimated glomerular filtration rate (eGFR)
  ✓ Cardiac function: echocardiography, electrocardiography (ECG), cardiac magnetic resonance imaging (CMRI)
  ✓ Neurological assessment: Brain magnetic resonance imaging (MRI), nerve conduction studies
  ✓ Ophthalmological examination
  ✓ Audiology assessment

For follow-up monitoring of FD, the following should be included:

- Regular follow-up visits (frequency depends on disease severity and treatment status)
- Periodic organ function assessments:
  ✓ Renal function: Every 6–12 months
  ✓ Cardiac function: Annually or more frequently if indicated
  ✓ Neurological assessment: Annually
  ✓ Ophthalmological and audiology examinations: Annually
- Biomarker monitoring:
  ✓ Lyso-Gb3 levels: To assess treatment response and disease progression
  ✓ hsTNT of TNI: For cardiac involvement
- Genetic counseling:
  ✓ Discuss inheritance pattern and implications for family members
  ✓ Genetic testing should be offered to at-risk relatives

Detailed guidance for initial evaluation and monitoring is discussed below according to specialty.

### 2.2. Cardiology

**Figure 1:**
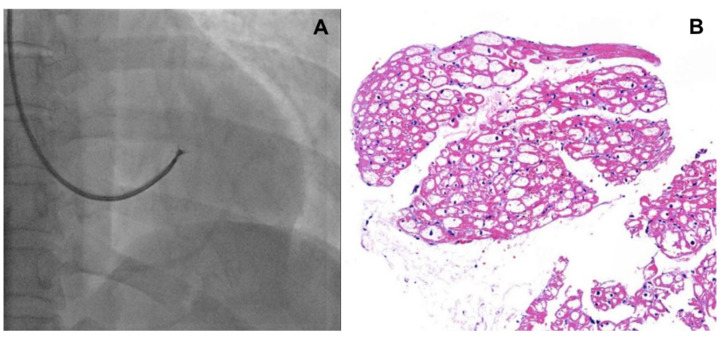
(**A**) Fluoroscopic image of endomyocardial biopsy, (**B**) H&E stain of endomyocardium, intracellular inclusions are found.

The prevalence of FD in patients with unexplained LVH ranges from 12 to 18% in highly selected cohorts, but most studies suggest a prevalence of approximately 0.5% to 1% in adult patients [6,7,8]. To evaluate cardiac function in patients with FD, various test modalities can be utilized:

- ECG and Holter monitoring: A short PR interval due to increased atrioventricular (AV) conduction without evidence of an accessory pathway is the earliest ECG feature in the subclinical stage of FD cardiomyopathy [9]. Voltage signs of LVH, strain pattern, and T-wave inversion in the precordial leads are the usual findings when overt FD cardiomyopathy has developed. In progressed overt FD cardiomyopathy, sinus bradycardia and advanced conduction abnormalities in the AV node/His bundle and distal conduction system are common and suggest an adverse prognosis [10]. Therefore, serial ECG follow-up and side-by-side comparison of ECGs are necessary for assessing disease progression. According to a study by Shah et al. published in 2005, non-sustained ventricular tachycardia (NSVT), defined as three or more consecutive ventricular premature beats at a rate of ≥100 beats per minute lasting less than 30 s, was a common finding on Holter monitoring in patients with Fabry disease—observed in 38% of males and 39% of females aged 60 years or older [11]. The prevalence of malignant ventricular arrhythmias increases with age and is associated with the extent of late gadolinium enhancement (LGE) on cardiac magnetic resonance imaging (CMRI) [12]. Although, as in hypertrophic cardiomyopathy, the relationship between NSVT and the risk of sudden cardiac death (SCD) remains unclear, prophylactic strategies for SCD should be considered in cases of NSVT detected in Holter monitoring, accompanied by LGE [7].
- Echocardiography is the most useful tool for diagnosing and monitoring cardiac involvement of FD. The typical findings are left ventricular (LV), right ventricular (RV), or papillary muscle hypertrophy, sometimes mimicking diffuse-type hypertrophic cardiomyopathy. However, asymmetric thickening of the interventricular septum or apical hypertrophy cannot exclude FD cardiomyopathy [7]. Myocardial strain and strain rate are useful for detecting early changes in non-overt FD cardiomyopathies. In treatment naïve patients with FD, early reduction of LV longitudinal strain (particularly basal segments) can develop without clear-cut LVH [13]. Valves can also be involved, as well as the myocardium. The mitral and aortic valves are often thickened, with mild-to-moderate regurgitation, whereas stenotic lesions are rare. As myocardial fibrosis develops during disease progression, paradoxical thinning and hypokinetic or akinetic movement of the involved segments can be observed. These findings suggest that severe and irreversible changes are associated with poor prognosis. According to a study conducted in Taiwan, 38.1% of males and 16.7% of females with late-onset FD had cardiac fibrosis, even without LVH [14]. The echocardiographic parameters that must be assessed are LV mass/mass index, velocity of the septal mitral annulus in early diastole (septal e’), ratio between early mitral inflow velocity and mitral annular early diastolic velocity (E/e’), maximal tricuspid regurgitation velocity (TR Vmax), right ventricular systolic excursion velocity (RV s’), tricuspid annular plane systolic excursion (TAPSE), pulmonary artery systolic pressure (PASP), global longitudinal strain (GLS), and isovolumic relaxation time (IVRT), in addition to routine parameters.
- CMRI provides an accurate assessment of LV size, mass, and geometry. The gadolinium contrast images visualize the localization and extent of myocardial fibrosis. The presence of extensive fibrosis is associated with a reduced response to ERT and an increased risk of arrhythmia [7]. CMRI can be used for differential diagnosis as well as for the prediction of prognosis. CMRI can also be used safely in patients with advanced chronic kidney disease and in patients undergoing hemodialysis. Native T1 mapping values (i.e., measured without contrast media such as gadolinium) are lower in patients with FD than in patients with normal myocardium, possibly due to sphingolipid accumulation [15].
- Although endomyocardial biopsy (EMB, Figure 1) is not recommended to determine treatment efficacy or to follow up cardiac involvement, it can be considered as a confirmatory diagnostic tool in patients with a variant of uncertain significance (VUS) or suspected cardiac variant of FD [7]. In addition, for patients who are not able to discontinue anticoagulation, EMB could be a good alternative for kidney biopsy. Lamellar bodies and intracellular inclusions found in electron microscopy provide strong evidence to diagnose FD.
- Coronary angiography: Damage to the coronary vascular bed by accumulation of glycolipids and secondary inflammations may lead to angina pectoris, variant angina, and myocardial infarction that do not necessarily follow the typical patterns observed in atherosclerosis [7]. Endothelial dysfunction with vasospasm and thrombotic events predominantly involves small, penetrating vessels. Therefore, angina symptoms in patients with FD need to be clarified by a coronary angiogram.

Follow-up of cardiac involvement in FD should be carried out regularly and the recommended schedule is based on outpatient visits every 3–6 months, as in Table 2.

### 2.3. Nephrology

Screening for Fabry nephropathy should focus on high-risk populations and involve both clinical evaluation and diagnostic testing. High-risk populations include individuals with unexplained proteinuria or chronic kidney disease of unknown origin, relatives of patients diagnosed with FD (especially male family members), and patients presenting with early-onset stroke or cardiomyopathy without identifiable causes [16].

Laboratory findings in urine are crucial for the diagnosis of FD. Urinalysis typically reveals proteinuria, often in the nephrotic range, along with possible microscopic hematuria and lipiduria [17]. Blood tests may indicate elevated serum creatinine levels and reduced eGFR. Low GLA enzyme activity in males strongly suggests FD. Imaging studies, such as renal ultrasound or magnetic MRI, may show enlarged kidneys in the early disease stages, progressing to atrophic kidneys as the disease advances [18]. Histopathological examination through renal biopsy can provide definitive evidence with characteristic findings like podocyte inclusions (zebra bodies) and glomerulosclerosis [19].

Effective monitoring of Fabry nephropathy necessitates the incorporation of specific biomarkers and assessment tools to assess disease progression and therapeutic response. In addition to the key biomarkers, including GLA activity and lyso-Gb3 levels, proteinuria and albuminuria serve as indicators of renal involvement, while serum creatinine and eGFR are crucial for evaluating renal function [20]. Assessment tools such as renal biopsy offer detailed histological insights, albeit invasively. Non-invasive imaging studies such as renal ultrasound and MRI aid in assessing kidney structure and identifying complications. Additionally, 24 h urine collection and regular urinalysis are essential for quantifying protein excretion and identifying renal pathology.

A structured visit schedule is vital for effective management. Initial evaluations at diagnosis should include comprehensive biomarker analysis and imaging studies. Routine follow-ups every 3 to 6 months, along with a thorough annual review, ensure continuous monitoring and timely adjustments to therapy [21]. This systematic approach enables healthcare providers to mitigate renal damage and enhance patient outcomes by closely monitoring disease progression and treatment response [21].

### 2.4. Neurology

The buildup of Gb3 disrupts normal endothelial function, contributing to a pro-inflammatory and pro-thrombotic state. This dysfunction can lead to vascular occlusions, increased vascular resistance, and a higher risk of ischemic events. Clinical manifestations that may raise suspicion and ultimately lead to the diagnosis of FD are as follows:

- Ischemic stroke: Ischemic strokes are more common in patients with FD than in the general population, often occurring at a younger age [22]. Strokes in Fabry patients are often due to small vessel disease, but larger vessel involvement and cardioembolism can also occur due to atrial fibrillation (AF) or other cardiac abnormalities related to FD [23].
- Transient Ischemic Attacks (TIAs): TIAs are characterized by brief episodes of neurological dysfunction caused by temporary ischemia, lasting less than 24 h. TIAs are often a warning sign of a future stroke and require immediate medical attention and preventive treatment.
- Cerebral small vessel disease: The accumulation of Gb3 in endothelial cells and smooth muscle cells of small blood vessels leads to their dysfunction and damage. This can result in thickening of the vessel walls, narrowing of the lumen, and reduced blood flow. These lesions are often detected incidentally on MRI scans such as white matter lesions and lacunar infarcts, even in asymptomatic patients [24]. They indicate chronic microvascular damage and are more prevalent in patients with FD. White matter lesions are associated with cognitive decline, memory impairment, and other neuropsychological deficits, contributing to the overall neurological burden in patients with FD.
- Cerebral large vessel disease: While small vessel disease is more common in FD, some patients may also present with large cerebral vessel involvement due to Gb3 accumulation. In some cases, these patients may develop stenosis, aneurysms, or dolichoectasia, or present with large vessel dissection, contributing to cerebrovascular events. Large vessel disease can lead to ischemic strokes from either atherosclerotic changes or emboli.
- Chronic neurologic symptoms: Patients may experience chronic headaches, dizziness, vertigo, and cognitive difficulties over time. Some patients may also present with silent strokes, which are small ischemic events that go unnoticed but can accumulate and lead to significant neurological impairment. Cognitive decline is common due to repeated ischemic events and the presence of white matter lesions. Patients may experience memory loss, difficulty concentrating, and other executive function deficits [25,26].

### 2.5. Gastroenterology

Gastrointestinal presentations in patients with FD are heterogeneous. Abdominal pain, diarrhea, constipation, bloating, vomiting, and nausea are common. Abdominal pain and diarrhea are the most common gastrointestinal symptoms [27]. The prevalence of gastrointestinal symptoms in patients with FD is unclear. Registry data in the Fabry Outcome Survey reported that 60% of untreated children and 50% of female adults had gastrointestinal symptoms [28,29].

The clinical features of FD are similar to those of functional gastrointestinal disorders. In addition, FD is rare, and non-specific gastrointestinal symptoms are often the initial presentation of the disease. Misdiagnosis due to non-specific gastrointestinal symptoms is common in patients with FD. Therefore, if FD is suspected, it is important to evaluate the patient’s history and determine the cause of the unexplained gastrointestinal symptoms. The presence of other FD-associated symptoms, including burning pain in the extremities, abnormal sweating, and heat intolerance, should raise suspicion of FD. Clinicians should evaluate the presence of other organ involvement, including the skin, eye, kidney, and heart, and assess the family history of FD-associated symptoms.

After diagnosis of FD, the 24 h and 7-day FABry Disease Patient-Reported Outcome-Gastrointestinal (FABPRO-GI) is a useful instrument for assessing and monitoring FD-related gastrointestinal symptoms in clinical trials and real-world settings [30]. Prokinetics, acid-suppressants, and loperamide are used to control gastrointestinal symptoms. Although no specific diet intervention was established as non-pharmacological therapy, a low Fermentable Oligosaccharides, Disaccharides, Monosaccharides, and Polyols (FODMAPs) diet is suggested as an effective alternative approach to control gastrointestinal symptoms in FD patients [31].

### 2.6. Otorhinolaryngology

A nationwide population-based study utilizing data from Taiwan’s National Health Insurance database showed that among patients diagnosed with FD, 16.7% experienced tinnitus, 7.5% had hearing disorders, and 1.7% suffered from sudden sensorineural hearing loss (SSNHL). These prevalence rates were significantly higher than those in the general control group [32]. Another study involving 57 patients with FD (mean age 46.2 years) found that 73.7% of patients exhibited SSNHL of ≥25 dB, 54.4% complained of dizziness, and 71.9% showed abnormal nystagmus responses [33]. Research focused on pediatric patients with FD reported tinnitus in 31.8–40% of patients, hearing loss in 12.8–21.8% of cases with onset around 4 years of age, and vertigo in 25.5–30.4% of cases with onset typically after 10 years of age; all symptoms occurred earlier in boys [34].

A thorough review of symptoms such as hearing loss, tinnitus, and dizziness is essential for newly diagnosed patients with FD and their family members who are suspected of having this condition. For a more systematic approach, validated questionnaires can be utilized, such as the Hearing Handicap Inventory for the Elderly [35], the Tinnitus Handicap Inventory [36], and the Dizziness Handicap Inventory [37,38].

After evaluating medical history, hearing tests should be conducted as part of the initial assessment [39]. A multidisciplinary management protocol for FD published in the Netherlands also recommends hearing tests in the initial examination [40]. For adult and pediatric patients with FD, the hearing tests that can be considered are detailed below:

- For adults: The initial otolaryngological hearing tests should include pure-tone audiometry (PTA), tympanometry, and Otoacoustic Emission (OAE) [39]. Depending on the patient’s reported symptoms, speech audiometry (SA) and tinnitogram (tinnitus pitch and loudness matching) can be added. PTA is the most widelyused subjective hearing test for assessinghearing thresholds across different frequencies. The OAE, particularly Distortion Product-OAE (DPOAE), is an objective hearing test that can detect early dysfunction of the outer hair cells in the cochlea. It can be used as a screening tool for patients with normal hearing who present with otological symptoms [41].
- For pediatric patients: PTA can be used reliably from 5 years of age onwards, under the guidance of a familiar audiologist. For younger children, visual reinforcement audiometry or play audiometry can be employed. However, these tests are subjective and may not always be accurate [42]. To overcome these limitations, objective hearing threshold measurements using DPOAE, Auditory Brain Response, and Auditory Steady State Response should be conducted.

Vestibular function tests are primarily conducted for patients reporting vestibular abnormalities and are not routinely recommended for initial assessment or periodic follow-up. For patients complaining of dizziness or vertigo, available tests include cranial nerve examination, nystagmus testing using electronystagmography or video nystagmography, positional and positioning tests, and video head impulse tests. For a more detailed examination of the peripheral vestibular organs, caloric tests and rotation chair tests can be performed.

There is no unified recommendation for regular monitoring of hearing in patients with FD. Some guidelines suggest conducting hearing tests when pediatric [34] or adult [40] patients complain of hearing loss. Conversely, other pieces of literature recommend annual follow-ups with PTA, tympanometry, and OAE testing [39]. For the regular monitoring of patients with FD, we recommend the following:

- Adult patients: For adults with normal hearing, there are no established principles for periodic hearing tests. However, hearing thresholds tend to increase with age [43], and hearing tests are recommended for adults with risk factors such as a family history of FD, genetic disorders associated with hearing loss, or noise exposure [44]. We recommend that patients with FD undergo periodic hearing tests for the early detection of hearing loss, even in the absence of reported symptoms.
- Pediatric patients: According to the newborn screening guidelines in South Korea, it is recommended that infants at high-risk for hearing loss should undergo regular otolaryngological examinations every 6 months to 1 year until school age, even if they pass the newborn hearing screening. These examinations should include an assessment of language development, otoscopy, and detailed hearing tests [45]. In line with these recommendations, we recommend that pediatric patients with FD undergo age-appropriate hearing tests at least once a year.
- Symptomatic patients: For patients complaining of dizziness or vertigo, we recommend conducting vestibular function tests as needed. In cases of sudden onset of symptoms such as SSNHL or acute vertigo, prompt otolaryngological consultations and appropriate treatment should be provided.

Table 3 summarizes the initial and follow-up assessments for otorhinolaryngological involvement in FD and recommends a schedule for outpatient visits.

### 2.7. Ophthalmology

Ocular manifestations of FD result from sphingomyelin accumulation within the lysosomes of eye tissues [46].

Vortex keratopathy (also called cornea verticillate), characterized by grayish or golden-brown deposits arranged in a vortex pattern in the corneal epithelial and subepithelial layers, is a common and diagnostically significant finding in patients with FD [46,47]. This manifestation occurs in most affected males and asymptomatic female carriers. Although it does not impact vision, the presence of vortex keratopathy is a highly sensitive and specific marker and crucial for early diagnosis of FD. It can be detected early and is present in about 70% of patients with FD. This condition can also occur due to amiodarone, chloroquine, indomethacin, and tamoxifen drug deposits, necessitating a systemic evaluation for differential diagnosis [47,48].

Another ocular manifestation of FD is the appearance of round intracellular inclusions in the conjunctival tissue, which may be accompanied by vascular changes such as tortuosity, microaneurysms, and dilation. These vascular changes can be seen in any conjunctival area but occur most commonly in the inferior bulbar area. Vessel tortuosity is more common in males than in females and has a significant correlation with the disease severity score [46,49].

Cataracts are the most common finding in the lens, particularly in the form of anterior capsular or posterior subcapsular opacities. Cataracts may have a detrimental effect on visual function, often requiring surgical intervention [46].

In the retina, the most common ocular finding is vascular tortuosity that is rarely accompanied by optic disc edema [46,49]. These changes can be detected by funduscopic examination.

**Figure 2:**
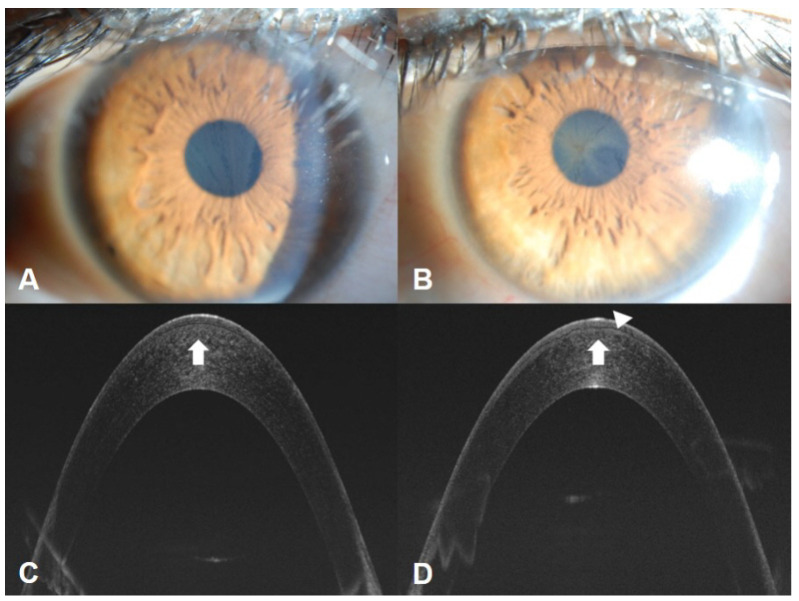
A 32-year-old woman with FD presented with decreased vision in her left eye, which had previously undergone Epi-LASIK. Photograph of the cornea showing the extent and density of vortex keratopathy or cornea verticillata, which was less significant in the right eye (**A**) compared to the left eye (**B**). Optical coherence tomography showed deposits (white arrows) in the anterior corneas of both eyes (**C**,**D**) and an irregular border between the corneal subepithelium and the anterior stroma layer (arrowhead, **D**). This suggests that prior refractive surgery may exacerbate corneal lesions in FD through altered wound healing.

Since the corneal wound healing process differs in FD, as shown in Figure 2., caution is required to prevent the progression of corneal opacity before and after surgery, such as Epi-LASIK. In FD, it is suspected that the wound healing process leads to intracellular Gb3 deposits in the surrounding tissue, exacerbating inflammation and opacity. Nevertheless, the specific processes causing the progression of corneal lesions are not known, necessitating further research.

### 2.8. Dermatology

Angiokeratomas are a representative skin symptom of FD [50]. They are reported in 70% of male patients and 39% of female patients [50], typically manifesting between the ages of 5 and 15 years in males, and between the ages of 8 and 25 years in females [29]. Angiokeratomas appear as dark red to bluish-black macules and papules ranging from pinpoint size to 4 mm in diameter, and their color does not blanch under pressure. Hyperkeratosis overlying the lesions varies and is rarely seen outside the genital and umbilical areas. They can be widespread or clustered. In males, angiokeratomas are mainly observed in the “bathing trunk” area, including the lower abdomen, umbilicus, groin, genitals, buttocks, and inner thighs. They are also commonly found on the proximal limbs, especially the medial aspects, and can appear in protruding areas such as the elbows and knees, as well as on the palms and soles, and the distal phalanges of digits. They can also be observed on the lips, particularly the vermilion border, and rarely on mucosal surfaces, but almost never on other parts of the face. In females, angiokeratomas are generally less common and can sometimes be found distributed in a dermatomal pattern. The most common areas are the trunk and proximal limbs, with genital lesions being relatively rare.

Histopathologically, angiokeratomas consist of dilated capillaries in papillary dermis surrounded by elongated rete ridges. Older lesions may exhibit varying degrees of hyperkeratosis. In skin lesions, lipid storage can be observed in endothelial cells, pericytes, arteriolar smooth muscle, and arrector pili muscle. These findings can be confirmed with periodic acid-Schiff or Sudan black B staining. Lipid accumulation may also be observed in perineural cells and eccrine gland epithelium.

Up to one-third of males and two-thirds of females do not present with angiokeratomas [51]. Some have no notable skin vascular lesions, while others have bright erythematous macular angiomatous lesions of 1–2 mm in diameter, which are thought to be early lesions of angiokeratomas [52]. There are also patients with widely distributed papular angiomas that clinically and histologically resemble cherry angiomas prevalent in up to 50% of the general population, making their clinical significance in FD unclear. Telangiectasias are also common and can be distinguished from angiokeratomas and angiomas by the loss of color under diascopy. They are reported more frequently in males (23%) than in females (9%). Telangiectasias appear later than angiokeratomas, with an average onset age of 26 years (range: 3–70 years) in males and 42 years (range: 5–73 years) in females. They are commonly observed in Caucasians and typically occur in sun-exposed areas such as the face and “V” area of the neck, but can also be seen on the flanks, groin, elbows, and knees. Dermoscopy reveals well-defined, dilated, and tortuous vessels in the upper dermis, particularly in patients with widely distributed angiokeratomas and a typical phenotype of the disease.

Hypohidrosis is a typical skin symptom of FD, which is thought to result from autonomic neuropathy, although structural accumulation in sweat glands may also play a role. In total, 53% of males and 28% of females reported reduced sweating, with earlier onset in males (average age: males 23 years, females 26 years). Anhidrosis was reported by 25% of males and 4% of females [53]. Heat intolerance, often accompanied by other symptoms, can lead to decreased exercise tolerance, nausea, dyspnea, dizziness, headaches, or loss of consciousness. Previous studies have confirmed improvement in sweating in patients with FD undergoing ERT [54]. Hyperhidrosis may occur more frequently in females than in males, with one study reporting the occurrence of hyperhidrosis in 44 of 369 females (11.9%) and 22 of 345 males (6.4%) [55].

### 2.9. Pathology

**Figure 3:**
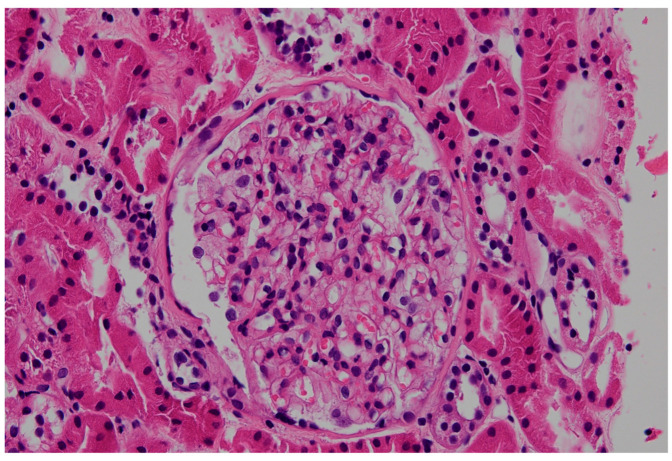
Podocytes and parietal epithelial cells show a vacuolated, honeycomb appearance resulting from accumulation of abnormal glycosphingolipid in Fabry disease and extraction of lipids during processing for standard light microscopy (hematoxylin and eosin, ×200).

In FD, podocyte cytoplasm appears expanded, foamy, pale, and lacy by light microscopy due to lipid deposits dissolved during processing of renal biopsy tissue [56,57] (Figure 3). With progressive disease, mesangial expansion and glomerulosclerosis develop, with proportional interstitial fibrosis and tubular atrophy. Segmental glomerular basement membrane double contours may be observed [56]. Inclusions are also present early in Henle’s loop and in the distal tubule, and occasionally in the proximal tubule [57]. Vascular sclerosis can be prominent even in early diseases. Standard immunofluorescence may show IgM and C3 in mesangial areas [56,57].

**Figure 4:**
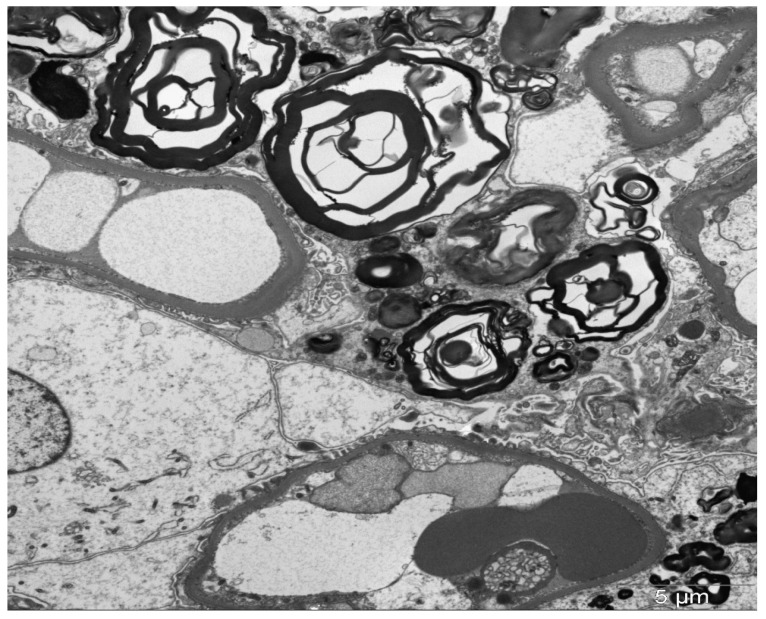
Lysosomal inclusion with a so-called “myelin body appearance” in the podocyte (transmission electron microscopy, ×12,000).

By electron microscopy (EM), inclusions in lysosomes are widespread and may be present in all renal cells. The processing of tissue for EM does not extract the lipids; therefore, the inclusions can be viewed directly on the toluidine blue-stained scout sections assessed for these studies. The inclusions vary in size and structure and have been called myelin bodies, whorled lamellated inclusions, or zebra bodies (Figure 4) [56,57]. Lamellated inclusions show alternating dark and light layers [56]. These inclusions are most prominent in podocytes but are also variably present in parietal epithelial cells, endothelium, peritubular capillaries, and interstitial cells [56]. Vascular smooth muscle cells also exhibit inclusions, with distal tubules frequently affected. Mosaic appearance of podocytes by EM, with lipid inclusions in some and none in other podocytes, may shed light on structural changes in affected versus nonaffected podocytes in female heterozygotes.

## 3. Diagnostic Challenges in Cases with a Variant of Uncertain Significance of the GLA Gene

Interpreting novel mutations and determining their pathogenicity can be problematic because of genetic complexity. While over 1000 *GLA* variants have been identified, many are classified as VUS, presenting significant diagnostic challenges. Screening may identify individuals with VUS or later-onset phenotypes, leading to diagnostic dilemmas [58]. High-throughput next-generation sequencing-based screening programs in high-risk populations and newborns have identified several novel *GLA* variants [59]. Understanding variant pathogenicity is the key to accurate prevalence estimation, diagnosis, and management of FD. Distinguishing disease-causing variants from benign bystanders is probably one of the biggest challenges in contemporary clinical genetics [59].

Screening studies have revealed a high prevalence of individuals with *GLA* genetic VUS. In newborn populations, the prevalence of *GLA* variants is approximately 0.04%, while in high-risk populations, it reaches 0.62% [60]. However, only a fraction of these cases (0.12% in high-risk populations) receive a definite diagnosis of classical FD [61].

Several factors contribute to the diagnostic challenges associated with VUS in FD:

- Heterogeneous clinical presentation: FD manifestations are highly variable, even among individuals with the same variant. This variability complicates the correlation between genotype and phenotype.
- Residual enzyme activity: Some VUS may result in partial enzyme activity, leading to milder or late-onset forms of the disease that are harder to diagnose.
- Lack of specific biomarkers: In some individuals with VUS, FD-specific biomarkers and imaging findings may not uncover evidence of organ involvement.
- Coexisting conditions: FD often coexists with other nephrotic disorders, such as IgA nephropathy, further complicating diagnosis [62].

To address these challenges, a comprehensive diagnostic approach including genetic tests, enzymatic activity assays, biomarker analysis, tissue biopsy, comprehensive clinical investigation, and family history and pedigree analysis is necessary. While essential, genetic testing alone is insufficient for the diagnosis of FD when a VUS is identified. Enzyme activity assays are crucial but may have varying cut-off values (10–55% of the mean reference value for men, and up to 80% for women). Biomarker analyses, including measurement of Gb3 or lyso-Gb3, can provide additional evidence, although levels may be normal in some VUS cases [63]. The kidney, skin, heart, or sural nerve biopsy can reveal characteristic features such as myeloid bodies, aiding in diagnosis. Furthermore, a thorough assessment of organ involvement, including renal, cardiac, gastrointestinal, and neurological examinations, is essential. Lastly, family history and pedigree assay can provide valuable context for interpreting VUS.

## 4. FD Treatment and Management of Associated Symptoms and Risk Factors

### 4.1. Genetics: ERT or Chaperone Therapy

There is currently no cure for FD, but treatments can alleviate symptoms, prevent progression, and improve patient quality of life [64,65]. Management involves a multidisciplinary approach with regular follow-ups with specialists, including nephrologists, cardiologists, neurologists, gastroenterologists, pain medicine specialists, and genetic counselors [34]. In South Korea, the management of FD encompasses both ERT and pharmacological chaperone therapy, tailored to the patient’s specific genetic mutation and clinical presentation.

ERT options comprise agalsidase alfa and agalsidase beta, administered every two weeks via intravenous infusion [34]. Clinical evidence has shown that these therapies are effective in stabilizing or improving renal function, reducing left ventricular mass index (LVMi), alleviating neuropathic pain, and enhancing overall quality of life [65,66,67,68,69,70,71]. Notably, early commencement of ERT is associated with improved outcomes, particularly in preserving organ function and delaying disease progression [72]. Adverse events are typically mild, with some patients experiencing infusion-related reactions that can be managed with premedication [73].

Recently, analyses of up to 20 years of data from the Fabry Outcome Survey (FOS) revealed that patients treated with Replagal (agalsidase alfa) experienced improvement or stabilization of renal, cardiac, morbidity, and mortality outcomes compared with untreated external Fabry disease cohorts [74]. Fabrazyme (agalsidase beta) has also been reported to reduce the risk of major clinical events (cardiac and cerebrovascular events) significantly compared to placebo over the last 20 years [75]. The Fabry Disease International Registry (Fabry Registry) obtained medical records from 1944 to 2002 for 447 patients from five countries, and also confirmed a preservation of kidney function and delays progression to severe clinical events among patients with FD following Fabrazyme treatment [76].

For patients aged 16 years and older with amenable mutations of the GLA gene, migalastat presents an oral treatment option. Clinical trials, such as the phase 3 ATTRACT study, have demonstrated that migalastat is comparable to ERT in maintaining renal function and reducing LVMi over a 30-month period [77,78]. Furthermore, migalastat improves gastrointestinal symptoms and stabilizes overall quality of life [71,77,79]. The safety profile of migalastat is favorable, with most adverse events being mild or moderate in severity [78]. However, its use is restricted to patients with specific amenable mutations [80] and in Korea, health insurance currently only covers reimbursement of migalastat after 12 months of ERT use.

For patients receiving either ERT or chaperone therapy, treatment adherence and tolerability should be assessed and monitoring for adverse effects is needed [80,81]. Treatment efficacy can be evaluated through improvement of clinical symptoms, change in biomarker levels (e.g., lyso-Gb3), and organ function tests [80].

### 4.2. Cardiology and Neurology

Given the overlapping and interrelated risk factors, an integrated and multidisciplinary approach to cardiovascular and neurologic risk factor management is critical in the care of patients with Fabry disease.

#### 4.2.1. Hypertension

Male patients with classical FD rarely have hypertension. If high blood pressure is measured at the outpatient clinic, consider 24 h blood pressure monitoring before starting treatment. Aim for a normal blood pressure of <130/80 mmHg in all patients according to current hypertension management guidelines [7,82]. Antihypertensive agents such as angiotensin-converting enzyme inhibitors (ACE inhibitors), angiotensin II receptor blockers (ARBs), and calcium channel blockers are commonly used, and more aggressive blood pressure control is often required after a stroke [83].

#### 4.2.2. Dyslipidemia

Low-density lipoprotein (LDL) and high-density lipoprotein (HDL) cholesterol are commonly elevated in FD. FD can be considered a high-risk disease in which endothelial dysfunction and vascular glycolipid accumulation develop frequently. While there are no official guideline recommendations, in our Fabry expert center, the risk for cardiovascular disease in patients with FD is considered similar to that of diabetes. Therefore, LDL cholesterol targets are stratified based on individual risk profiles: <100 mg/dL for young patients (<50 years) without additional risk factors, <70 mg/dL for those with comorbidities such as hypertension or smoking, and <55 mg/dL for patients with established atherosclerotic cardiovascular or cerebrovascular disease. Statins are the primary lipid-lowering agents, with high-intensity therapy recommended for both primary and secondary prevention [84].

#### 4.2.3. Coexisting Diabetes

In patients with coexisting diabetes, which significantly increases the risk of ischemic stroke, strict glycemic control is critical. A target HbA1c level of <7% is advised, achieved through lifestyle modification and pharmacologic therapy such as metformin or insulin [84].

#### 4.2.4. Atrial Fibrillation (AF)

While not common in all FD patients, AF may occur particularly in those with cardiomyopathy or advanced age [7]. If AF is detected, stroke prevention with anticoagulation is indicated. In patients with non-valvular AF, direct oral anticoagulants (DOACs)—including dabigatran, rivaroxaban, apixaban, and edoxaban—are preferred due to superior safety profiles and ease of use [83,85]. Warfarin remains the treatment of choice in patients with valvular AF, such as those with moderate-to-severe mitral stenosis or mechanical heart valves, with an INR target of 2.0–3.0 [83].

#### 4.2.5. Antiplatelet Therapy

In patients with a history of ischemic stroke, antiplatelet therapy such as aspirin or clopidogrel is essential for secondary prevention, with short-term dual therapy considered in certain subtypes [83,85]. For cardioembolic strokes, anticoagulation therapy is mandatory. Furthermore, in patients with significant carotid artery stenosis, carotid endarterectomy or stenting may be considered to prevent recurrent stroke [83].

### 4.3. Nephrology

Numerous studies have shown that ERT with agalsidase α or agalsidase β can stabilize renal function in patients with Fabry nephropathy, especially when initiated early in disease progression [86]. While ERT and chaperone therapy are effective, additional treatments such as renin–angiotensin–aldosterone system inhibitors are beneficial once kidney damage has occurred. Since ERT alone has not been shown to reduce proteinuria consistently, complementary treatments like angiotensin-converting enzyme inhibitors and angiotensin receptor blockers are vital for managing proteinuria in Fabry nephropathy patients [87]. Newer drugs, such as sodium-glucose cotransporter-2 inhibitors, hold promise as additional therapies for Fabry nephropathy.

### 4.4. Gastroenterology

Certain medications can provide symptom improvement for specific gastrointestinal symptoms. The use of metoclopramide showed symptom relief in some patients with gastroparesis [88]. Proton pump inhibitors are used to improve upper gastrointestinal symptoms. Anti-diarrhea medications such as loperamide may be useful to control lower gastrointestinal symptoms. Probiotics and antibiotics can be a potential treatment to prevent or control bacterial overgrowth [89]. The use of gabapentin has demonstrated a beneficial effect in patients with neuropathic pain [90].

### 4.5. Otorhinolaryngology

In patients with FD with multi-organ involvement, otolaryngological symptoms can be easily overlooked by both patients and healthcare providers. However, these symptoms significantly impact patients’ quality of life and, if left untreated at the appropriate time, can lead to a greater burden on the patient. It affects not only communication and language development but also cognitive function, educational attainment, employment prospects, and mental health. Hearing loss can lead to social isolation, loneliness, and a reduced quality of life. In children, it can impair speech and language development, while in adults, it is associated with an increased risk of dementia [91]. For patients with FD who are at risk of progressive hearing loss, timely and appropriate management of hearing impairment is crucial to mitigate these potential adverse effects and maintain overall quality of life.

Tinnitus can significantly impact various aspects of a person’s life. It affects listening and communication, often making it difficult to understand speech, especially in noisy environments. In children, tinnitus can impair language and speech development. Educationally and professionally, it can lead to reduced performance and lower achievement. Tinnitus frequently contributes to social isolation and loneliness, particularly in women and older adults. Mental health can be adversely affected, with higher rates of depression and lower quality of life reported among tinnitus sufferers [92].

There is currently no clear evidence that ERT can improve auditory symptoms [93]. Appropriate follow-up and testing through collaboration among various departments are necessary, and immediate specialist consultation is recommended when related symptoms occur [34,39]. Symptomatic treatment is provided for hearing loss, tinnitus, and vertigo, depending on the situation. Hearing rehabilitation for patients with hearing loss most commonly involves the use of hearing aids, while cochlear implants may be considered for severe hearing loss [39].

Furthermore, in patients with FD with multi-organ involvement and a predisposition to cerebrovascular and cardiovascular diseases, the possibility of acute emergencies such as SSNHL or central vertigo should always be considered, necessitating immediate intervention when required. SSNHL is, in particular, an otological emergency requiring prompt diagnosis and appropriate treatment [94]. Similarly, vertigo cases may require appropriate testing and differential diagnosis between peripheral and central vertigo to guide treatment.

### 4.6. Pain Management

Neuropathic pain is one of the most debilitating manifestations of FD, significantly impacting the quality of life of affected individuals [95]. Current strategies for managing neuropathic pain in patients with FD are discussed here.

A recent survey found that frequently used acute pain medications among patients with FD include non-steroidal anti-inflammatory drugs (NSAIDs) and non-opioid analgesics, with few patients using neuropathic pain-control agents [96]. This could be because there are limited comparative clinical data for the use of neuropathic pain-control agents specifically in patients with FD. Nevertheless, case reports of small numbers of patients with FD have shown that pain can be reduced with the administration of carbamazepine, gabapentin, phenytoin, and intravenous lidocaine.

For chronic pain management, current pain-management strategies are founded on clinical experience or based on national and international guidelines for the management of neuropathic pain. Based on a systematic literature review and meta-analysis of pharmacotherapy for the management of neuropathic pain in adults, tricyclic antidepressants, serotonin and norepinephrine reuptake inhibitors (e.g., duloxetine, venlafaxine), carbamazepine, and gabapentinoids (e.g., gabapentin, pregabalin) should be considered as first-line options. Intravenous lidocaine, topical capsaicin (8%) patches, and tramadol should be considered as second-line options. Strong opioids should be considered as third-line options, with controlled-release opioids being preferred over short-acting opioids. Fourth-line treatments may include methadone (with both N-methyl-D-aspartate and opioid receptor effects), tapentadol, and anticonvulsants with lesser evidence of efficacy (e.g., lamotrigine, lacosamide) [96,97,98,99,100,101].

Tricyclic antidepressants and serotonin–norepinephrine reuptake inhibitors enhance the levels of neurotransmitters that inhibit pain signals, providing relief from neuropathic pain [102]. Carbamazepine and phenytoin work by stabilizing the inactivated state of sodium channels, thereby reducing neuronal excitability and decreasing the transmission of pain signals [103]. This makes them effective options for treating neuropathic pain, which is characterized by abnormal nerve function and heightened pain sensitivity. The use of carbamazepine in FD-related neuropathic pain is supported by its efficacy in treating other forms of neuropathic pain, such as trigeminal neuralgia and diabetic neuropathy [104]. While specific studies evaluating carbamazepine for FD are limited, the drug’s established role in managing neuropathic pain suggests it can be beneficial for patients with FD experiencing similar symptoms. There is some evidence to suggest that phenytoin may be useful during pain attacks [105].

Gabapentinoids such as gabapentin and pregabalin are commonly used to manage neuropathic pain in FD. These medications work by modulating calcium channels and reducing neuronal excitability, thereby alleviating pain. Clinical trials have demonstrated their efficacy in reducing pain severity in patients with FD [99].

Lidocaine and capsaicin plasters may be useful to control localized pain, as well as during pain crises. However, the mostly episodic nature of pain in FD reduces the appropriateness of these drugs. Furthermore, in FD, the area affected by pain crises is too large for plasters. Based on clinical experience, lidocaine or capsaicin cream may be used as acute-pain prophylaxis before physical activity. There is some evidence to suggest that phenytoin may be useful during pain attacks [96,105]. More recently, intravenous lidocaine has been used to successfully treat FD pain crisis [99].

Opioids may be considered for severe neuropathic pain that is unresponsive to other treatments. However, their use is generally limited due to the risk of dependence and side effects. Controlled-release opioids are preferred over short-acting opioids. Opioids may also be effective during pain crises, but to avoid worsening gut motility problems and to reduce the risk of dependency, they should be used only when anticonvulsant agents are ineffective. A multimodal approach, combining opioids with other pain management strategies and close patient monitoring, is essential to optimize pain relief while minimizing risks [106].

Key characteristics of neuropathic pain control agents specifically in patients with FD are summarized in Table 4.

Ketamine has been used to effectively treat pain with a neuropathic component; however, because of its adverse events and potential addiction risks, its use should be restricted to therapy-resistant severe neuropathic pain until definite proof is obtained that its benefits outweigh the risks and costs of treatment [107]. Although the potential of alpha-2 adrenoceptor agonists for the management of chronic neuropathic pain has also been explored, further clinical trials in this area are probably required [108].

Because of the distinct phenotype and comorbidities in patients with FD and since evidence for the use of analgesic treatment in FD is scarce, individualized recommendations will be necessary in most cases.

A multidisciplinary approach is important when forming an analgesic care plan, involving not only metabolic and pain specialists but also specialist pharmacists, pain psychologists, and physiotherapists. The management of pain in FD is challenging due to the unique interplay between several factors. Neuropathic pain, which is normally challenging to treat, occurs with multi-organ dysfunction, often limiting pharmacological management. Additionally, the most severe symptoms are often found in males starting in their youth, a time of significant psychosocial development.

## 5. Discussion

*Evidence supports the role of a multidisciplinary team approach in managing Fabry disease*

There are currently no randomized controlled trials (RCTs) specifically designed to evaluate the effectiveness of the MDT model in Fabry disease. Most evidence supporting MDT care comes from expert consensus, registry data, and observational studies rather than from RCTs [109]. The MDT approach has been demonstrated to provide better outcomes in terms of disease stability after ERT and clinical regression, including patients with mild organ impairment [110]. In addition, a recent study in a Chinese hospital highlighted that the MDT assessment allowed for the identification of FD in 35 high-risk children, whereas none were identified before the establishment of the MDT [111].

*Our experiences of the MDT approach for FD diagnosis and management*

MDTs are characterized by two distinct models. The first, a concurrent consultation model, involves all specialists convening simultaneously for a unified patient assessment. This approach offers heightened efficiency, expedited inter-professional communication, enhanced educational opportunities, and a reduction in overall patient consultation time. The second, a “quarterback” physician model, features a designated primary physician who coordinates consultations with organ-specific specialists, who must be designated team members. While this model may extend the patient’s overall consultation time due to sequential departmental visits, it minimizes individual specialist consultation time, thereby facilitating MDT implementation by lowering the barrier to initial team formation. Both models necessitate a comprehensive understanding of FD among all team members and require regular inter-specialist meetings and communication.

**Figure 5:**
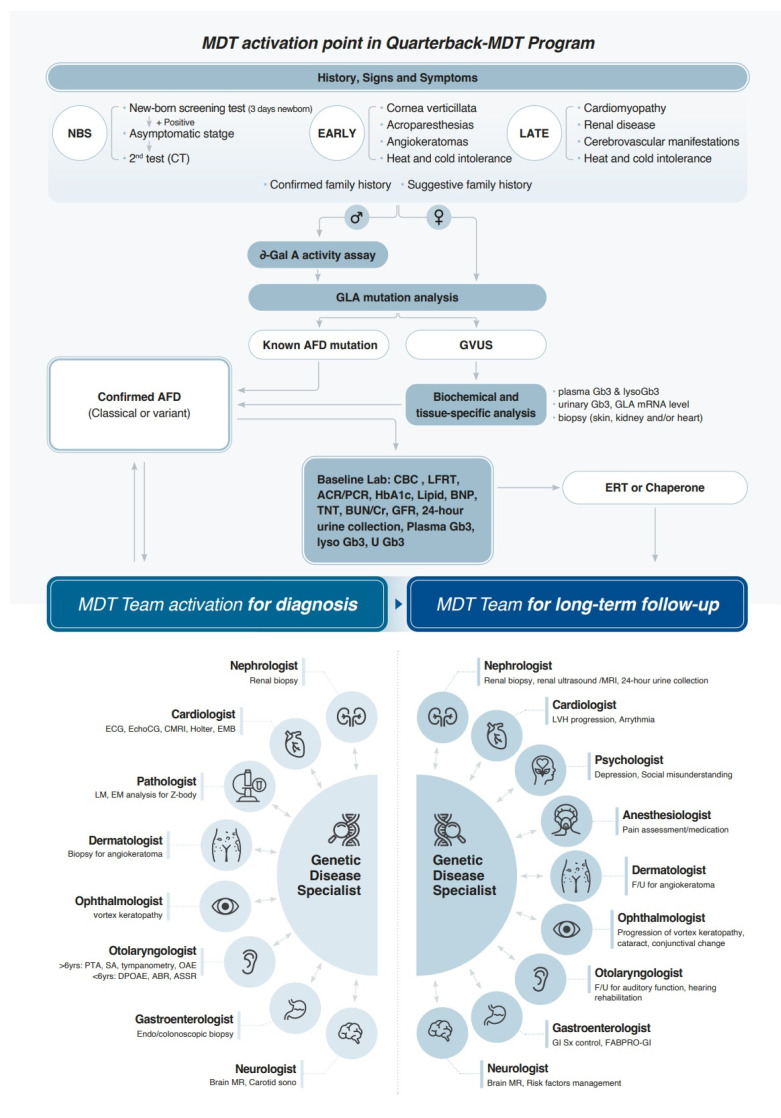
MDT activation points in Quarterback-MDT program for Fabry disease. MDT: multidisciplinary team; NBS: newborn screening; GLA: alpha-galactosidase A; AFD: Anderson–Fabry disease; GVUS: genetic variants of unknown significance; Gb3: globotriaosylceramide; lysoGb3: globotriaosylsphingosine; mRNA: messenger ribonucleic acid; CBC: complete blood count; LFRT: liver function test; ACR/PCR: albumin/protein-to-creatinine ratio; HbA1c: hemoglobin A1c; BNP: brain natriuretic peptide; TNT: troponin T; BUN: blood urea nitrogen; Cr: creatinine; GFR: glomerular filtration rate; U: urine; ERT: enzyme replacement therapy; ECG: electrocardiography; EchoCG: echocardiography; CMR: cardiac magnetic resonance imaging; EMB: endomyocardial biopsy; LM: light microscopy; EM: electron microscopy; PTA: Pure Tone Audiometry; SA: Speech Audiometry; OAE: Otoacoustic Emission; DPOAE: distortion product OAE; ABR: auditory brainstem response; ASSR: auditory steady-state response; MR: magnetic resonance imaging; sono: sonography; LVH: left ventricular hypertrophy.

Our team, recognizing the pragmatic advantages of the latter model within the South Korean healthcare landscape, initiated a collaborative effort in 2021. This involved five specialties engaging in regular case discussions pertaining to diagnosis and treatment, thereby fostering team development. By 2024, the team had evolved into an FD Center of Excellence (COE) comprising individual specialists from ten disciplines. This expanded team convenes for biannual to triannual meetings, facilitating discussions, peer education, and the exploration of optimized treatment strategies. Figure 5 schematically illustrates our quarterback system. The genetic specialist assumes the role of the primary physician for FD patients, spearheading initial diagnosis and ERT. MDT activation occurs at diagnosis and during follow-up, with variations in involved specialties. Following confirmatory diagnosis and ERT initiation, follow-up intervals for participating specialties range from 3 to 6 months, tailored to individual patient disease status.

While the MDT model for FD faces substantial barriers in resource-limited areas, these can be mitigated through targeted training, telemedicine and remote MDT, international partnerships, advocacy for national health policy change, and the gradual introduction of emerging therapy. Early diagnosis and coordinated care remain critical to improving outcomes for FD patients everywhere.

### 5.1. Future Direction for Improved Diagnosis and Management of VUS Cases

In cases where patients with variants of uncertain significance (VUS) do not meet current treatment indications, identifying multi-organ involvement and treatment needs in Fabry disease underscores the essential role of the multidisciplinary team (MDT) in diagnosis and management. Since Fabry disease symptoms may not manifest simultaneously across all organ systems—and in some cases may progress more severely in a specific organ—long-term monitoring by an MDT is crucial to determining the pathogenicity of VUS. Over time, such monitoring can help elucidate the clinical significance of these variants. Functional research, including both in vitro and in vivo studies assessing the impact of VUS on enzyme activity and cellular processes, will further support early diagnosis and intervention. Additionally, data sharing and collaboration—not only within institutions and at the national level but also internationally—are vital for building robust evidence on rare variants.

### 5.2. Areas of Improvement in Reimbursement Criteria of FD Treatment in South Korea

According to the current Korean reimbursement guideline (Table 1), the cost for ERT cannot be reimbursed for patients with cardiac variants who do not meet the LVH criteria (LV wall thickness > 12 mm) despite a clear family history and genotype. However, subtle histologic and echocardiographic changes (shown by tissue Doppler image or longitudinal strain) are known to start before the development of overt LVH in treatment-naive patients with FD [13,82,112]. Frustaci et al. proved that in the prehypertrophic stage (left ventricular maximal wall thickness ≤ 10.5 mm), ERT stabilizes storage deposits and myocyte dimensions regardless of sex [113]. In the study by Langeveld et al. investigating the optimal timing of ERT initiation, starting ERT before the age of 16 years in male patients with classic FD reduced the occurrence of renal and cardiac manifestations [114]. Therefore, it is necessary to refine reimbursement conditions so that ERT can be started early if the genotype is typical, thereby improving long-term prognosis.

In South Korea, the reimbursement criteria for ERT (Table 1) for renal management of FD necessitates the presence of proteinuria and include the following conditions: a reduced eGFR (15 ≤ eGFR < 90 mL/min/1.73 m^2^, adjusted for age > 40) measured on at least two occasions; microalbuminuria detected more than twice over a minimum 24 h interval and albumin > 30 mg/g (in males); albuminuria detected more than twice over a minimum 24 h interval and albumin > 20 µg/min (in males); proteinuria > 150 mg/24 h (in males); and proteinuria > 300 mg/24 h with clinical evidence of progression (in females).

However, our previous research showed that segmental foot process effacement and Gb3 deposits can persist in Fabry nephropathy despite ERT [19]. Additionally, these deposits are observed in various kidney cell types in normoalbuminuric patients with FD, demonstrating the limitations of using albuminuria alone to detect early renal damage. These findings highlight the need for baseline and follow-up kidney biopsies for the appropriate initiation and monitoring of ERT in Fabry nephropathy. Therefore, we suggest that the reimbursement criteria for ERT should also account for the presence of Gb3 deposits in kidney biopsy specimens, even in normoalbuminuric patients with FD.

To contextualize Korea’s reimbursement framework within a broader global perspective, Table 5 summarizes ERT eligibility criteria across representative countries. While Korea offers a clearly structured and standardized approach, its strict organ-damage thresholds—such as requiring overt proteinuria or LVH—may limit timely access to treatment, particularly for female patients or those with late-onset phenotypes. These individuals may not meet reimbursement criteria despite histopathological or imaging evidence of progressive disease. In contrast, many international guidelines have evolved toward preventive treatment strategies, recommending ERT initiation based on confirmed genotype, early signs, or tissue-level involvement. Such approaches aim to optimize long-term outcomes through earlier intervention. These international trends highlight the potential benefit of adopting more flexible, genotype- and biomarker-driven reimbursement models in Korea.

MDT members must recognize the importance of not only vital organ problems but also symptoms from other functional areas, including the intestine, eyes, ears, and skin, that significantly affect the quality of life and overall health of patients with FD. A group of specialists gathered as an MDT should conduct regular assessments and monitoring in various clinical specialties, and a prompt and effective interdisciplinary consultation system should be implemented to address possible emergencies such as sudden hearing loss or central vertigo. Regular MDT meetings for collaborative clinical decisions and education will greatly contribute to improving health management and quality of life for patients with FD and thus deserve financial and operational support at the national level.

To complement the discussion in Section 5.2, Table 5 presents a cross-national comparison of ERT reimbursement systems for Fabry disease, focusing on South Korea, Japan, Australia, and select European countries. While South Korea’s reimbursement criteria are highly structured and organ-damage-oriented, they are considered relatively restrictive. Patients often require overt clinical manifestations—such as reduced eGFR, proteinuria, or established left ventricular hypertrophy (LVH)—to initiate ERT, making early intervention difficult, especially in female or late-onset patients [1]. In contrast, Australia provides subsidized ERT through the Life Saving Drugs Program (LSDP), which permits treatment based on histological or imaging findings of organ involvement and includes chronic pain or ischemic vascular events as eligible criteria. However, the process requires reapplication and clinical data submission every year to maintain eligibility [2]. European countries, including Italy and the UK, largely follow the EMA indication for Replagal^®^, reimbursing ERT for patients with confirmed Fabry diagnosis regardless of sex or phenotype. These systems emphasize the importance of early intervention and broader access, with fewer bureaucratic restrictions [3,4,5]. Japan’s practical guidelines recommend ERT initiation in classical male patients upon the onset of characteristic symptoms such as acroparesthesia. For late-onset male and female patients, ERT is advised when there is clear evidence of organ involvement, confirmed by regular monitoring of renal, cardiac, and neurologic parameters [6]. This international landscape underscores the need for Korean reimbursement policies to evolve toward a more genotype-driven and biomarker-guided model, aligning with global trends favoring early and preventive treatment strategies.

## 6. Conclusions

The diagnosis and long-term follow-up of FD, a multi-organ systemic disorder, necessitate an MDT approach to ensure high-quality management. Given the rarity of FD, it is unrealistic to expect all physicians to possess comprehensive knowledge of this condition. Therefore, PNUYH has established an MDT, which has been operational for over 4 years and comprises designated specialists from each relevant department who possess a thorough understanding of FD. This team is coordinated by a “quarterback” physician who maintains a comprehensive overview of each patient’s condition and medical history. The team has progressively expanded over time, and the sophistication of case discussions has significantly improved, enabling the successful management of numerous challenging cases, including those involving VUS. It is anticipated that this review report on FD diagnosis and management from an MDT perspective will serve as a valuable resource for centers initiating MDTs for the optimized management of FD.

## Figures and Tables

**Table 1:** Reimbursement criteria for enzyme replacement therapy (ERT) in South Korea [5].

**Eligibility criteria for ERT reimbursement of patients with FD**		
Patients must meet both of the following criteria: Patients demonstrating decreased GLA activity in leukocytes or skin fibroblasts and confirmed to have FD through genetic testing. In female patients where no reduction in GLA activity is observed, a definitive diagnosis can still be established based on genetic testing. Patients presenting with clinical symptoms or signs consistent with FD that fulfill at least one of the nine criteria listed below, with other potential causes excluded through differential diagnosis.		
Category	Requirements	
Nephrology *	1. Decreased estimated glomerular filtration rate (15 ≤ eGFR < 90 mL/min/1.73 m^2^; adjusted for age > 40 years) detected twice or more	
	Male	2. Microalbuminuria (>30 mg/g) (detected twice or more over a minimum interval of 24 h)
		3. Albuminuria (>20 μg/min) (detected twice or more over a minimum interval of 24 h)
		4. Proteinuria (>150 mg/24 h)
	Female	5. Proteinuria with clinical evidence of progression (>300 mg/24 h)
Cardiology	6. Left ventricular hypertrophy (left ventricular wall thickness > 12 mm) diagnosed with magnetic resonance imaging or echocardiography (however, in patients with hypertension, at least 6 months of blood pressure treatment should be performed prior to administration of this drug), etc.	
	7. Clinically significant arrhythmias and conduction disturbances, etc.	
Neurology	8. Stroke or TIA confirmed by objective examination, etc.	
Pain	9. Chronic, uncontrolled neuropathic pain despite the use of antiepileptic drugs and/or maximum dose analgesics (NSAIDs, etc.) (the drug effect must be continuously proven through medical records, etc., for continuous administration)	
**Assessment of ERT effectiveness**		
Before initiating ERT, patients should undergo a comprehensive baseline evaluation. The therapeutic efficacy of ERT should be assessed periodically through renal function tests (e.g., eGFR) or cardiac function tests (e.g., ECG) at intervals of 6 to 12 months to monitor treatment response and disease progression.		

* In the case of kidney-related requirements, a confirmatory diagnosis through biopsy is recommended for differential diagnosis from other causes.

**Table 2:** Recommended tests and schedules of cardiac evaluations in classical and non-classical types of Fabry disease.

Cardiac Examination	Classical	Non-Classical (Cardiac Variant)
B-type natriuretic peptide, ST2	At initial assessment Every 6–12 months	At initial assessment Every 6–12 months
Lipid profiles	At initial assessment Every 6–12 months	At initial assessment Every 6–12 months
Electrocardiogram	At initial assessment Every 6–12 months	At initial assessment Every 6–12 months
24 h Holter	At initial assessment Depending on symptoms	At initial assessment Depending on symptoms
Treadmill test	At initial assessment Every 12 months	At initial assessment Every 12 months
Echocardiography	At initial assessment Every 12 months	At initial assessment Every 12 months
Exercise echocardiography	Depending on symptoms	Depending on symptoms
CMRI (including T1 mapping, late gadolinium enhancement)	At initial assessment Every 3–5 years	At initial assessment Every 3–5 years
Coronary angiography	Depending on symptoms (angina)	Depending on symptoms (angina)

**Table 3:** Recommended tests and schedules of otorhinolaryngological evaluations in Fabry disease patients.

Otorhinolaryngological Examination	Diagnosis and Follow-Up
PTA, SA	At initial assessment (for patients > 6 years old) Follow-up every 12 months
DPOAE	At initial assessment Follow-up every 12 months
Tympanometry	At initial assessment Follow-up every 12 months
Tinnitogram	Depending on symptoms
ABR, ASSR	At initial assessment (for patients < 6 years old, if DPOAE result was abnormal; and for patients > 6 years old, if they show signs of auditory neuropathy) Follow-up every 12 months (if earlier ABR or ASSR was abnormal)
Cranial nerve examination, Nystagmus test, Positional and positioning tests, Video head impulse test	Depending on symptoms

**Table 4:** Recommended analgesic drugs for supportive treatment of chronic neuropathic pain in Fabry disease.

Agents	Mechanism of Action	Dose	Side Effects
**First line**			
Tricyclic antidepressants - Amitriptyline - Nortriptyline	Serotonin and norepinephrine reuptake inhibition. Action on dopaminergic pathways and locus coeruleus.	12.5–150 mg/day	Dry mouth, sedation, arrythmias, urinary retention, diarrhea, cognitive disturbance, worsening of autonomic instability
Serotonin and noradrenaline reuptake inhibitors	Serotonin and norepinephrine reuptake inhibition		Serotonergic syndrome, gastrointestinal discomfort, diarrhea, anxiety, dizziness
- Duloxetine		- 60–120 mg/day	
- Venlafaxine		- 150–225 mg/day	
Carbamazepine	Reduced sodium channel conductance. Reduction in ectopic discharges.	250–800 mg/bid	Associated with blood dyscrasias, Stevens–Johnson syndrome, toxic epidermal necrolysis, hyponatremia
Phenytoin		300 mg/day	
Gabapentinoids	Inhibit calcium-mediated neurotransmitter release through effects on *α*2δ-1 subunits. NMDA receptor antagonism.		Weight gain, cognitive dysfunction, lethargy
- Gabapentin		- Titrated from 100 mg tid to 3600 mg tid	
- Pregabalin		- Starting dose 50 mg bid up to 300 mg bid	
**Second line**			
Intravenous lidocaine	Local anesthetic causing sodium channel blockade.	2–5 mg/kg	Local anesthetic systemic toxicity
Topical capsaicin (8%) patches	Depletion of substance P	0.0125% applied topically for 12 h daily	Burning, pruritus
Tramadol	Serotonin and norepinephrine reuptake inhibitor. μ-opioid receptor agonist.	100–400 mg/day	May lower seizure threshold
**Third line**			
Strong opioids	Opioid receptor agonists.		Nausea, constipation, itching, respiratory depression, osteoporosis, reduced immunity, endocrine dysfunction
- Morphine		- 30–120 mg q12h	
- Oxycodone		- 20–60 mg q12h	
**Fourth line**			
Methadone	NMDA antagonist activity Norepinephrine reuptake inhibition and μ-opioid receptor agonist.	50 mg bid maximum 500 mg/day	Nausea, constipation, itching, respiratory depression, osteoporosis, reduced immunity, endocrine dysfunction
Tapentadol	Sodium channel blockade and suppressed release of glutamate.	25 mg/day for 2 weeks up to a maximum of 400 mg/day.	Anxiety, anorexia asthenia, diarrhea, heat or cold intolerance, gastrointestinal discomfort, muscle spasms, sleep disorders, tremor
Less efficacious anti-convulsants (Lamotrigine, lacosamide)			Aggression, agitation, arthralgia, diarrhea, dizziness, drowsiness, dry mouth, fatigue, headache, irritability, nausea, pain, rash, sleep disorders, tremors, vomiting

Bid—twice daily; tid—three times daily; q12h—12 hourly.

**Table 5:** International comparison of reimbursement criteria for enzyme replacement therapy (ERT) in Fabry disease.

Country	Summary of Reimbursement Criteria for ERT	Key Features
South Korea	- Confirmed GLA gene variant - At least one of 9 FD-related clinical manifestations (nephropathy, cardiomyopathy, neuropathic pain, stroke, etc.) - Nephrology: eGFR 15–90 mL/min/1.73 m^2^ (age-adjusted > 40 years), proteinuria > 150 mg/24 h (males), >300 mg/24 h (females) - Cardiology: LV wall thickness > 12 mm (after ≥6 months of BP control if hypertensive) - Neurology: History of stroke or TIA - Pain: Chronic refractory neuropathic pain	- Strict organ-damage-based reimbursement - Early-stage or subclinical patients (e.g., females, late-onset males) face limited access
Australia	- Confirmed diagnosis of Fabry disease via enzyme assay or GLA mutation - At least one of the following: proteinuria, LVH, ischemic event, or chronic refractory pain - Renal/cardiac biopsy or imaging (e.g., MRI) to confirm organ damage recommended - Requires initial application and annual reapplication demonstrating clinical benefit	- ERT subsidized via Life Saving Drugs Program (LSDP) - Focus on organ involvement and documented treatment effect - Evidence of effectiveness needed for ongoing eligibility
Europe (Italia and UK)	- ERT approved for long-term use in patients with confirmed diagnosis of Fabry disease - Diagnosis based on enzymatic deficiency or GLA mutation - Indication-based reimbursement across most EU countries	- No strict organ damage requirement - Aligns with EMA labeling and early intervention recommendations - More inclusive of females and early-stage patients
Japan	- Confirmed GLA gene mutation - Classic males: Initiate ERT upon symptom onset (e.g., acroparesthesia) - Late-onset males and females: ERT when evident organ damage present - Recommended monitoring (q6–12 months): proteinuria, GFR, ECG, echocardiography, brain MRI	- Genotype- and symptom-driven reimbursement in classic males - Organ damage required for females and late-onset phenotypes - Structured national monitoring protocol

Abbreviations: ERT: Enzyme Replacement Therapy; FD: Fabry Disease; GLA: Galactosidase Alpha; LVH: Left Ventricular Hypertrophy; LV: Left Ventricle; eGFR: Estimated Glomerular Filtration Rate; BP: Blood Pressure; TIA: Transient Ischemic Attack; LSDP: Life Saving Drugs Program (Australia); MRI: Magnetic Resonance Imaging; ECG: Electrocardiogram; EMA: European Medicines Agency.

## Abbreviations

The following abbreviations are used in this manuscript:

AHA	American Heart Association
ASA	American Stroke Association
CEA	Carotid endarterectomy
CMRI	Cardiac magnetic resonance imaging
DPOAE	Distortion Product Otoacoustic Emission
ECG	Electrocardiogram
EMB	Endomyocardial biopsy
FD	Fabry disease
Gb3	Globotriaosylceramide
eGFR	estimated glomerular filtration rate
GLA	Alpha-galactosidase A
HbA1c	Glycated hemoglobin
HDL	High-density cholesterol
hsTNT	High-sensitive troponin T
LDL	Low density lipoprotein
LV	Left ventricular
LVH	Left ventricular hypertrophy
MDT	Multidisciplinary team
MRI	Magnetic resonance imaging
NSAIDs	Non-steroidal anti-inflammatory drugs
OAE	Otoacoustic Emission
PNUYH	Pusan National University Yangsan Hospital
PTA	Pure Tone Audiometry
RV	Right ventricular
SA	Speech Audiometry
TIA	Transient ischemic attack
VUS	Variants of uncertain significance
